# The combined influence of multiple sex and growth hormones on risk of postmenopausal breast cancer: a nested case-control study

**DOI:** 10.1186/bcr3040

**Published:** 2011-10-21

**Authors:** Shelley S Tworoger, Bernard A Rosner, Walter C Willett, Susan E Hankinson

**Affiliations:** 1 Channing Laboratory, Department of Medicine, Brigham and Women's Hospital and Harvard Medical School, 181 Longwood Avenue, Boston, MA 02115, USA; 2 Department of Epidemiology, Harvard School of Public Health, 677 Huntington Avenue, Boston, MA 02115, USA; 3 Department of Biostatistics, Harvard School of Public Health, 677 Huntington Avenue, Boston, MA 02115, USA; 4 Department of Nutrition, Harvard School of Public Health, 677 Huntington Avenue, Boston, MA 02115, USA

## Abstract

**Introduction:**

Sex and growth hormones are positively associated with postmenopausal breast cancer risk. However, few studies have evaluated the influence of multiple hormones simultaneously.

**Methods:**

We considered the roles of estrone, estradiol, estrone sulfate, testosterone, androstenedione, dehydroepiandrosterone (DHEA), DHEA sulfate and prolactin and, secondarily, insulin-like growth factor 1 (IGF-1) and c-peptide in postmenopausal breast cancer risk among 265 cases and 541 controls in the prospective Nurses' Health Study. We created several hormone scores, including ranking women by the number of hormones above the age- and batch-adjusted geometric mean and weighting hormone values by their individual associations with breast cancer risk.

**Results:**

Women in the top versus bottom quintile of individual estrogen or androgen levels had approximately a doubling of postmenopausal breast cancer risk. Having seven or eight compared to zero hormones above the geometric mean level was associated with total (RR = 2.7, 95% CI = 1.3 to 5.7, *P *trend < 0.001) and estrogen receptor (ER)-positive (RR = 3.4, 95% CI = 1.3 to 9.4, *P *trend < 0.001) breast cancer risk. When comparing the top versus bottom quintiles of the score weighted by individual hormone associations, the RR for total breast cancer was 3.0 (95% CI = 1.8 to 5.0, *P *trend < 0.001) and the RR for ER-positive disease was 3.9 (95% CI = 2.0 to 7.5, *P *trend < 0.001). The risk further increased when IGF-1 and c-peptide were included in the scores. The results did not change with adjustment for body mass index.

**Conclusions:**

Overall, the results of our study suggest that multiple hormones with high circulating levels substantially increase the risk of breast cancer, particularly ER-positive disease. Additional research should consider the potential impact of developing risk prediction scores that incorporate multiple hormones.

## Introduction

Circulating levels of estrogens and androgens are positively associated with the risk of breast cancer in postmenopausal women [1-4]. Furthermore, growth hormones, including prolactin and insulin-like growth factor 1 (IGF-1), are modestly associated with risk [5,6]. However, relatively little is known about the possible combined effect of multiple hormones simultaneously with respect to risk [1,2].

Several studies have assessed the joint effects of sex and growth hormones on postmenopausal breast cancer risk [2,5,7,8], and, in general, no multiplicative interactions were observed. However, Yu *et al. *[7] reported that women with high levels of IGF-1 and at least one sex hormone had the highest risk of breast cancer. For example, compared to women with low IGF-1 and low testosterone levels, those with high levels of both had a RR = 2.2 (95% CI = 1.0 to 4.5) versus a RR = 1.7 (95% CI = 0.8 to 3.6) for high testosterone only or a RR = 1.1 (95% CI = 0.5 to 2.4) for IGF-1 only. Only one small study (29 cases and 58 controls) considered more than two hormones at the same time [9]. Estrone, estradiol, androstenedione, dehydroepiandrosterone sulfate (DHEAS), testosterone and IGF-1 were measured on prospectively collected serum samples. Women whose hormone levels were below the age-adjusted mean for all hormones had a substantially lower risk of breast cancer compared to women with one or more hormone levels above the mean (RR = 0.11, 95% CI = 0.01 to 0.90). The authors suggested that mammotropic hormones may act as permissive factors for breast cancer growth.

To assess this hypothesis and to extend the concept of developing hormone scores, we evaluated the role of multiple hormones on postmenopausal breast cancer risk using data from 265 patients and 541 controls in the prospective Nurses' Health Study (NHS) who had measures of estrone, estradiol, estrone sulfate, testosterone, androstenedione, dehydroepiandrosterone (DHEA), DHEAS and prolactin. We created a hormone score similar to that described by Trichopoulos *et al. *[9], as well as scores based on other approaches, including weighting hormone values by their individual association with breast cancer risk or by their proliferative effect on breast cancer cell lines. On the basis of prior data indicating that sex hormones tend to be more strongly associated with estrogen receptor (ER)-positive disease [2,5,6], we evaluated the scores for this subset of tumors. Secondarily, we considered a subset of women who also had IGF-1 and c-peptide measures.

## Materials and methods

### Study population

The NHS cohort was established in 1976 among 121,700 US female registered nurses ages 30 to 55 years. All women completed an initial questionnaire and have been followed biennially by questionnaire to update exposure status and disease diagnoses. In 1989 and 1990, 32,826 NHS participants (ages 43 to 69 years) provided blood samples and completed a short questionnaire [10]. Briefly, these women arranged to have their blood drawn and shipped with an ice pack via overnight courier to our laboratory, where it was processed and separated into plasma, red blood cell and white blood cell components. These samples have been stored in liquid nitrogen freezers since collection. This study was approved by the Committee on the Use of Human Subjects in Research at Brigham and Women's Hospital in Boston.

The women were diagnosed with breast cancer after blood collection but before 1 June 1998. The follow-up rate was 99% in 1998, and the maximum follow-up time was 8.83 years. We confirmed 320 breast cancer patients who were postmenopausal and not using postmenopausal hormones (PMH) at the time of blood collection. Cases were matched to two controls on age (± 2 years), month of blood collection (± 1 month), time of day of blood draw (± 2 hours) and fasting status. The Institutional Review Board of Brigham and Women's Hospital approved this analysis, and all participants provided their written informed consent.

Among the potentially eligible 320 cases and 663 controls, 41 cases and 79 controls were missing data for one hormone, 10 cases and 21 controls were missing data for two hormones, and 4 cases and 22 controls were missing data for three or more hormones. In total, 265 cases (147 ER-positive cases) and 541 controls had assay values for all estrogens, androgens and prolactin. A subset (*n *= 182 cases and *n *= 370 controls) also had IGF-1 and c-peptide data.

### Laboratory assays

Hormone assay methods have been described previously [10,11] and were conducted by four different laboratories. Estrone, estradiol, androstenedione, testosterone, DHEA and DHEAS were assayed at the Quest Diagnostics Nichols Institute (San Juan Capistrano, CA, USA) using an extraction step (except for DHEAS) followed by RIA. For estrone sulfate, the first batch was assayed at the University of Massachusetts Medical Center's Longcope Steroid Radioimmunoassay Laboratory (Worcester, MA, USA). The remaining batches were assayed at the Nichols Institute. To quantify estrone sulfate levels, estrone was first extracted from the plasma and then the estrone sulfate bond was enzymatically cleaved to release estrone, which was extracted from the plasma by an organic solvent and studied by chromatography and RIA [12]. The first two batches of sex hormone-binding globulin (SHBG) and prolactin were assayed at the Longcope Laboratory. The remaining samples were assayed at Massachusetts General Hospital's Reproductive Endocrinology Unit Laboratory (Boston, MA, USA). Prolactin was measured by microparticle enzyme immunoassay and SHBG was analyzed using the AxSYM immunoassay system (Abbott Diagnostics, Chicago, IL, USA). All assays for IGF-1 and c-peptide were conducted at the laboratory of Dr Michael Pollak at McGill University (Montreal, QC, Canada) using an ELISA from Diagnostic Systems Laboratory (Webster, TX, USA). Free estradiol and testosterone were calculated according to the method described by Sodergard *et al. *[13].

Cases and matched controls identified through 1 June 1998 were assayed for estrogens, androgens and prolactin. Cases and matched controls identified through 1 June 1996 were assayed additionally for IGF-1 and c-peptide. Case-control sets were assayed together, ordered randomly and labeled to mask case-control status. The coefficient of variation from blinded replicate samples was less than 15% for all assays. When hormone values were less than the detection limit, we set the value to half this limit. The detection limits of the assays and the number of samples below the limit were 2 pg/ml estradiol (*n *= 2), 10 pg/ml estrone (*n *= 22), 40 pg/ml estrone sulfate (*n *= 7), 5 ng/dl androstenedione (*n *= 3), 2 ng/dl testosterone (*n *= 4), 10 ng/dl DHEA (*n *= 1), 5 μg/dl DHEAS (*n *= 5), 0.6 ng/ml prolactin (*n *= 0), 0.09 ng/ml IGF-1 (*n *= 0) and 0.02 ng/ml c-peptide (*n *= 0).

### Statistical analysis

We identified statistical outliers using the generalized extreme studentized deviate many-outlier detection approach [14]. This resulted in the removal of the following values: four estradiol (≥76 pg/ml), three testosterone (≤1 or ≥459 ng/dl), three androstenedione (≤5 ng/dl), two DHEA (≤14 ng/dl), five DHEAS (≤5 μg/dl), six prolactin (≥43 ng/ml), two IGF-1 (≤24 ng/ml) and one c-peptide (≤0.03 ng/ml). Furthermore, some assays could not be conducted because of low sample volume or technical difficulties with the assay.

We used unconditional logistic regression models to estimate RR and 95% CI for total breast cancer, invasive breast cancer and ER-positive disease, adjusting for matching factors, including age at blood collection (continuous), fasting status (yes or no) and time of day of blood draw (1 to 8 AM, 9 AM to noon or 1 PM to midnight). All analyses were adjusted for age at menopause (continuous), parity (continuous), family history of breast cancer (yes or no) and personal history of benign breast disease (yes or no). Secondary analyses further adjusted for body mass index (BMI) at the time of blood draw.

We estimated the association of each hormone individually with postmenopausal breast cancer risk. Hormones were modeled in quintiles (based on cutpoints in control women), and RRs compared the top and bottom quintile. Because of the relatively high correlations between sex hormones (mean = 0.36, range = 0.13 to 0.71, 40% > 0.40), the models were unstable when we included multiple estrogens, androgens or all hormones simultaneously in the same multivariate model. As such, a stepwise approach to evaluating the addition of hormones to the statistical model was not feasible. Therefore, we created several scores to combine information about all hormones; these are described below. The primary hormone scores included the three estrogens, four androgens and prolactin. The secondary analyses also included IGF-1 and c-peptide. We calculated each score for estrogens only and androgens only and included the two scores in the same statistical model to evaluate the independent associations of these two important hormone classes. Scores were categorized as indicated in Tables 2 through 4 and were included as a continuous term to calculate tests for trend using the Wald test.

The first goal of this study was to replicate the analysis by Trichopoulos *et al. *[9], thus we created a score that was the sum of the number of hormones for each woman above the age- and batch-adjusted mean level. The results derived using the median were similar and therefore are not shown. We were interested in expanding the concept of developing a hormone score, as the above score does not take into account that some hormones are more strongly associated with risk than others. To account for the varying strengths of the association across hormones, we created a score that weighted each hormone by its individual association with overall breast cancer risk on the basis of the following equation:

$$Score_{1}=\overset{h}{\underset{m=1}{\sum }}\beta_{m}*\log_{2}\left(X_{m}\right),$$

where *m *stands for each of the measured *h *hormones, β_m _is the coefficient for that hormone modeled linearly on the log_2 _scale with breast cancer risk and *X*_m _is the value of the hormone. We used β coefficients for hormone levels on the log_2 _scale modeled continuously that were reported in the literature with the largest sample size evaluating sex hormones [1], prolactin [5] and IGF-1 [8] (Tim Key for the Endogenous Hormones and Breast Cancer Collaborative Group, personal communication for continuous β on the log_2 _scale) and our own data for c-peptide, as a pooled analysis was unavailable. The coefficients used were 0.372 for estrone, 0.255 for estradiol, 0.239 for estrone sulfate, 0.344 for testosterone, 0.293 for androstenedione, 0.215 for DHEA, 0.174 for DHEAS, 0.182 for prolactin, 0.166 for IGF-1 and 0.108 for c-peptide.

We also were interested in considering the biological influence of the hormones on breast cancer cells and thus created a score that weighted each hormone on the basis of a proliferation index (PI_m_) with respect to MCF-7 breast cancer cells, which is a common cell model of breast cancer. We used this metric because biological data were available for all hormones, at physiological concentrations, using this cell line and measuring some marker of proliferation. Since assay metrics that have been published to measure proliferation differed across hormones, we calculated PI_m _by dividing the cell proliferation results for each hormone by the cell proliferation results for estradiol under the same conditions (for example, same assay, molar concentration and starting cell number) and calculated the score by using the following equation:

$$Score_{2}=\overset{h}{\underset{m=1}{\sum }}PI_{m}*\ln\left(X_{m}\right),$$

where *m *stands for each of the measured *h *hormones, PI_m _is the proliferation index based on published data and *X*_m _is the value of the hormone. The PI_m _values (minimum and maximum) were 1.0 for estradiol (reference), 0.75 (0.51 to 1.10) for estrone [15-19], 0.45 (0.40 to 0.50) for estrone sulfate [19,20], 0.60 (0.21 to 0.78) for testosterone [21-26], 0.49 (0.35 to 0.67) for androstenedione [26-29], 0.72 (0.40 to 1.08) for DHEA [20,23,24,26], 0.82 (0.78 to 0.85) for DHEAS [20,23,24] and 1.04 (0.88 to 1.25) for prolactin [25,30,31]. All *P *values were based on two-sided tests and were considered statistically significant if P ≤ 0.05. The analyses were conducted using SAS version 9.1 software (Cary, NC, USA).

## Results

The mean age of cases was 62.1 years (SD = 4.5) and that of controls was 61.8 years (SD = 4.7). A history of benign breast disease was more common in cases than in controls (37.7% vs 30.1%), as was a family history of breast cancer (20.4% vs 15.3%). The mean age at menopause was 50.2 years (SD = 3.6) in cases and 50.8 years (SD = 3.8) in controls. Mean parity was 3.0 (SD = 1.9) in cases and 3.3 (SD = 2.0) in controls.

In a multivariate models including standard breast cancer risk factors, women with estrogen or androgen levels in the top versus bottom quintile had approximately a doubling of postmenopausal breast cancer risk (RR range = 1.5 to 2.5) (Table 1). For most of the sex hormones, the association was qualitatively stronger for ER-positive disease than for total breast cancer. Prolactin was suggestively associated with ER-positive breast cancer risk (RR = 1.7), but associations for IGF-1 and c-peptide were not statistically significant. These results are consistent with prior reports from the NHS [2,5,6].

**Table 1:** Hormone levels among postmenopausal women not taking hormones in the Nurses' Health Study and the individual association between each hormone and breast cancer risk overall and for estrogen receptor-positive tumors

	Median (10th to 90th percentile)		RR, top vs bottom quintile (95% CI)^a, b^	
	
Hormones	Cases	Controls	All cases	ER-positive cases
Estrone (pg/ml)	28 (15 to 49)	24 (14 to 43)	2.1 (1.3 to 3.4)	2.8 (1.5 to 5.3)
Estradiol (pg/ml)	8 (4 to 17)	6 (4 to 14)	2.4 (1.4 to 4.1)	2.9 (1.4 to 5.9)
Estrone sulfate (pg/ml)	276 (102 to 733)	222 (98 to 577)	2.4 (1.5 to 3.9)	2.2 (1.2 to 4.0)
Testosterone (ng/ml)	24 (14 to 49)	22 (12 to 40)	1.8 (1.1 to 2.9)	2.0 (1.0 to 3.7)
Androstenedione (ng/ml)	63 (35 to 108)	57 (30 to 108)	2.1 (1.3 to 3.6)	2.6 (1.3 to 5.0)
DHEA (ng/dl)	234 (104 to 506)	218 (93 to 435)	1.5 (0.9 to 2.4)	1.6 (0.9 to 2.9)
DHEAS (μg/dl)	92 (41 to 224)	85 (33 to 175)	2.5 (1.4 to 4.2)	2.0 (1.0 to 3.8)
Prolactin (ng/ml)	8.2 (4.9 to 14.8)	8.0 (4.9 to 14.0)	1.1 (0.7 to 1.7)	1.7 (0.9 to 3.1)
IGF-1 (ng/ml)	152 (98 to 252)	153 (98 to 242)	1.1 (0.6 to 2.0)	1.4 (0.7 to 2.7)
c-peptide (ng/ml)	1.71 (0.66 to 4.11)	1.53 (0.61 to 4.03)	1.4 (0.8 to 2.4)	1.4 (0.7 to 3.0)

DHEA = dehydroepiandrosterone; DHEAS = dehydroepiandrosterone sulfate; ER = estrogen receptor; IGF-1 = insulin-like growth factor 1. ^a^RR and 95% CI were adjusted for age at blood draw and menopause, parity, history of benign breast disease, family history of breast cancer, fasting status and time of day of blood draw. ^b^For all hormones (except IGF-1 and c-peptide), *n *= 265 total cases (invasive and *in situ*), *n *= 147 ER-positive, invasive cases, and *n *= 541 controls; for IGF-1 and c-peptide, *n *= 182 total cases (invasive and *in situ*), *n *= 105 ER-positive, invasive cases, and *n *= 370 controls.

Increasing numbers of hormones above the age- and batch-adjusted geometric mean level were linearly associated with total (RR per one-unit increase = 1.16, 95% CI = 1.08 to 1.24, *P *trend < 0.001) and ER-positive (comparable RR = 1.19, 95% CI = 1.09 to 1.29, *P *trend < 0.001) breast cancer risk (Table 2). Comparing those data with seven or eight hormones above the geometric mean level versus none, the RR was 2.7 (95% CI = 1.3 to 5.7) for breast cancer overall and 3.4 (95% CI = 1.3 to 9.4) for ER-positive disease. Because of the small number of ER-positive cases with no hormones above the geometric mean level, we also compared those with seven or eight hormones above the geometric mean versus zero or one hormone and observed that the RR was 3.0 (95% CI = 1.5 to 5.9). A similar linear association with risk was observed for the score weighted by individual hormone associations. Women in the top versus the bottom quintile had a RR = 3.0 (95% CI = 1.8 to 5.0, *P *trend < 0.001) for total breast cancer and 3.9 (95% CI = 2.0 to 7.5, *P *trend < 0.001) for ER-positive tumors. In the score weighted by the PI_m_, women in the top versus bottom quintile had a 2.5-fold increased breast cancer risk and a 3.5-fold increased risk of ER-positive disease. Scores developed using the minimum or maximum PI_m _had similar associations and had a correlation of 0.99 with the score using the mean PI_m _(data not shown). Adjustment for BMI at the time of blood draw did not substantially change the results (data not shown). For example, the RR comparing the top versus the bottom quintile of the score weighted by individual hormone associations, after adjusting for BMI, was 2.9 (95% CI = 1.7 to 4.8) for total breast cancer and 3.7 (95% CI = 1.9 to 7.2) for ER-positive disease. The three hormone scores were very highly correlated (range = 0.90 between the score summing the number of hormones above the geometric mean and the score weighting by the PI_m _to 0.98 between the score weighting by the breast cancer association and that weighting by PI_m_).

**Table 2:** Risk of breast cancer among postmenopausal women not using hormones in the Nurses' Health Study on the basis of several hormone scores^a^

	Sample size			RR (95% CI)^b^	
	
Hormone scores	Total cases	ER+ cases	Controls	All cases	ER+ cases^c^
Number of hormones > geometric mean^d^					
0	11	5	43	1.0	1.0
1 to 2	35	18	113	1.1 (0.5 to 2.4)	1.2 (0.4 to 3.4
3 to 4	63	35	146	1.6 (0.8 to 3.3)	1.8 (0.7 to 5.0)
5 to 6	82	43	137	2.1 (1.0 to 4.5)	2.4 (0.9 to 6.5)
7 to 8	74	46	102	2.7 (1.3 to 5.7)	3.4 (1.3 to 9.4)
Per 1-unit increase	265	147	541	1.16 (1.08 to 1.24)	1.19 (1.09 to 1.29)
Per 1 SD increase^d^	265	147	541	1.42 (1.21 to 1.66)	1.51 (1.24 to 1.85)
*P *trend	265	147	541	< 0.001	< 0.001
Score weighted by individual hormone associations^e^					
Quintile 1	30	14	109	1.0	1.0
Quintile 2	42	24	107	1.4 (0.8 to 2.4)	1.7 (0.8 to 3.5)
Quintile 3	48	25	109	1.6 (0.9 to 2.7)	1.8 (0.9 to 3.7)
Quintile 4	58	32	108	2.0 (1.2 to 3.3)	2.3 (1.1 to 4.5)
Quintile 5	87	52	108	3.0 (1.8 to 5.0)	3.9 (2.0 to 7.5)
Per 1 SD increase^e^	265	147	541	1.45 (1.23 to 1.70)	1.54 (1.26 to 1.88)
*P *trend	265	147	541	< 0.001	< 0.001
Score weighted by MCF-7 proliferation^f^					
Quintile 1	35	16	108	1.0	1.0
Quintile 2	37	27	109	1.0 (0.6 to 1.7)	1.6 (0.8 to 3.2)
Quintile 3	55	23	108	1.6 (0.9 to 2.6)	1.5 (0.7 to 2.9)
Quintile 4	53	29	109	1.5 (0.9 to 2.5)	1.8 (0.9 to 3.5)
Quintile 5	85	52	107	2.5 (1.5 to 4.1)	3.5 (1.8 to 6.6)
Per 1 SD increase^f^	265	147	541	1.44 (1.22 to 1.69)	1.55 (1.26 to 1.89)
*P *trend	265	147	541	< 0.001	< 0.001

ER = estrogen receptor. ^a^Hormones included in the score are estrone, estradiol, estrone sulfate, testosterone, androstenedione, dehydroepiandrosterone, dehydroepiandrosterone sulfate and prolactin. ^b^RR and 95% CI were adjusted for age at blood draw and menopause, parity, history of benign breast disease, family history of breast cancer, fasting status and time of day of blood draw. ^c^*n *= 147 ER-positive cases. ^d^Score is the number of hormones for which the participant had higher than the age- and assay batch-adjusted geometric mean hormone level. Mean = 4.0 (SD = 2.4). ^e^Score is the sum of the log_2_-transformed hormone level multiplied by the β coefficient for that hormone with breast cancer risk. Mean = 10.8 (SD = 1.1) among controls. ^f^Score is the sum (across all hormones) of the natural log-transformed hormone level multiplied by a standardized proliferation rate (compared to estradiol) for MCF-7 breast cancer cells. Mean = 20.2 (SD = 2.1) among controls.

The results were similar for invasive breast cancer and when using free estradiol and free testosterone instead of total levels, although the sample size was decreased because of missing SHBG levels (data not shown). The associations between the hormone scores and breast cancer risk also were similar after excluding cases diagnosed in the first two years of follow-up and when stratifying by age (younger than 65 years of age vs age 65 years or older) or BMI (less than 25 kg/m^2 ^vs 25 kg/m^2 ^or more) (data not shown). Each score was qualitatively more strongly associated with breast cancer risk among women who had never used PMH versus past users, although none of the interactions were statistically significant (data not shown), likely because one assessment of hormone levels better reflects long-term exposure in women who have never used PMH.

When a separate score for estrogens and androgens was included in the same statistical model, the association for the estrogen score was slightly stronger than for the corresponding androgen score, although the associations were not statistically significantly different (*P *= 0.52) (Table 3). For example, the RR comparing the top versus bottom quintile for the estrogen score weighted by individual hormone associations was 2.1 (95% CI = 1.2 to 3.7, *P *trend = 0.005) and that for the androgen score was 1.7 (95% CI = 1.0 to 3.0, *P *trend = 0.06) for all breast cancers. The comparable associations for ER-positive disease were 2.4 (95% CI = 1.2 to 4.9, *P *trend = 0.003) and 2.0 (95% CI = 1.0 to 4.2, *P *trend = 0.21), respectively. Adjustment for BMI at the time of blood draw did not appreciably change the risk estimates for total breast cancer or ER-positive disease (data not shown). The correlation between the estrogen and androgen scores was 0.41 for the score summing the number of hormones above the geometric mean and 0.47 for the score weighted by the association with breast cancer risk.

**Table 3:** Risk of breast cancer among postmenopausal women not using hormones in the Nurses' Health Study on the basis of several estrogen and androgen scores^a^

	RR (95% CI)^b^, all cases^c^		RR (95% CI)^b^, ER+ cases^c^	
	
Hormone scores	Estrogen scores	Androgen scores	Estrogen scores	Androgen scores
Number of hormones > geometric mean^d^				
0	1.0	1.0	1.0	1.0
1	1.5 (1.0 to 2.5)	1.1 (0.6 to 2.0)	1.4 (0.8 to 2.6)	1.2 (0.6 to 2.5)
2	1.5 (0.9 to 2.3)	1.4 (0.8 to 2.5)	1.6 (0.9 to 2.8)	1.8 (0.9 to 3.5)
3 or 4	1.9 (1.3 to 3.0)	1.5 (0.9 to 2.3)	2.2 (1.3 to 3.8)	1.5 (0.8 to 2.6)
Per 1-unit increase	1.21 (1.06 to 1.38)	1.12 (1.00 to 1.25)	1.29 (1.09 to 1.53)	1.09 (0.95 to 1.25)
Per 1 SD increase^§^	1.25 (1.07 to 1.47)	1.18 (1.00 to 1.40)	1.36 (1.11 to 1.67)	1.14 (0.92 to 1.40)
*P *trend	0.01	0.05	0.003	0.22
Score weighted by individual hormone associations^e^				
Quintile 1	1.0	1.0	1.0	1.0
Quintile 2	1.2 (0.7 to 2.1)	1.1 (0.7 to 2.0)	1.2 (0.6 to 2.5)	1.2 (0.6 to 2.6)
Quintile 3	1.8 (1.1 to 3.0)	1.2 (0.7 to 2.1)	1.7 (0.9 to 3.4)	1.8 (0.9 to 3.6)
Quintile 4	1.3 (0.7 to 2.2)	1.2 (0.7 to 2.1)	1.3 (0.6 to 2.6)	1.2 (0.6 to 2.5)
Quintile 5	2.1 (1.2 to 3.7)	1.7 (1.0 to 3.0)	2.4 (1.2 to 4.9)	2.0 (1.0 to 4.2)
Per 1 SD increase^e^	1.28 (1.08 to 1.52)	1.19 (0.99 to 1.42)	1.38 (1.12 to 1.71)	1.16 (0.92 to 1.46)
*P *trend	0.005	0.06	0.003	0.21

ER = estrogen receptor. ^a^Hormones included in the score are estrone, estradiol, estrone sulfate in the estrogen score and testosterone, androstenedione, dehydroepiandrosterone, dehydroepiandrosterone sulfate in the androgen score. ^b^RR and 95% CI were adjusted for age at blood draw and menopause, parity, history of benign breast disease, family history of breast cancer, fasting status and time of day of blood draw. ^**c**^*n *= 265 total cases and *n *= 147 ER+ cases. ^d^Score is the number of hormones for which the participant had higher than the age- and assay batch-adjusted geometric mean hormone level. Among controls, the estrogen score mean = 1.5 (SD = 1.2) and the androgen score mean = 2.1 (SD = 1.5). ^e^Score is the sum of the log_2_-transformed hormone level multiplied by the β coefficient for that hormone with breast cancer risk. Among controls, the estrogen score mean = 4.3 (SD = 0.6) and the androgen score mean = 6.0 (SD = 0.7).

No interactions were observed between the estrogen and androgen scores (*P *heterogeneity > 0.33). When we considered women with both estrogen and androgen scores below the 75th percentile (4.7 for the estrogen score and 6.4 for the androgen score) versus those with both scores above the 75th percentile, there was a suggestion of a combined effect for total breast cancer. Compared to women with low estrogen and androgen scores, those with a high estrogen score only had a RR = 1.8 (95% CI = 1.2 to 2.8), those with a high androgen score only had a RR = 1.4 (95% CI = 0.9 to 2.2) and those who had high scores for both hormones had a RR = 2.1 (95% CI = 1.3 to 3.2). The results were similar for ER-positive tumors (data not shown).

When we examined the score weighted by the individual hormone associations, we observed that the RR for a 1 SD increase was similar for the score considering the sex hormones and prolactin (RR = 1.50) and after adding the IGF-1 and c-peptide scores (RR = 1.53) (Table 4). However, when we considered the number of hormones above the geometric mean, the RR increased from 2.2 to 3.2 for women with more than eight hormones versus those with zero or one hormone after adding IGF-1 and c-peptide. Furthermore, women who had hormone levels above the age- and batch-adjusted geometric mean for all ten hormones (versus zero or one) had the highest risk of breast cancer (RR = 4.8, 95% CI = 1.5 to 15.8).

**Table 4:** Risk of breast cancer among postmenopausal women not using hormones in the Nurses' Health Study by several mammotropic hormone scores^a ^among 182 cases and 370 controls

	RR (95% CI)^b^			
	
Mammotropic hormone score	Cases/controls, *n*	Sex hormones and prolactin	Cases/controls, *n*	Adding IGF-1 and c-peptide
Number of hormones > geometric mean^c^				
0 or 1	20/65	1.0	8/40	1.0
2 or 3	25/90	0.9 (0.5 to 1.8)	25/74	1.7 (0.7 to 4.1)
4 or 5	52/99	1.6 (0.9 to 3.0)	36/92	1.9 (0.8 to 4.5)
6 or 7	64/87	2.3 (1.2 to 4.2)	64/92	3.2 (1.4 to 7.3)
8+	21/29	2.2 (1.0 to 4.9)	49/72	3.2 (1.4 to 7.5)
Per 1-unit increase	182/370	1.17 (1.07 to 1.26)	182/370	1.15 (1.06 to 1.23)
Per 1 SD increase^c^	182/370	1.44 (1.19 to 1.75)	182/370	1.38 (1.16 to 1.66)
*P *trend	182/370	< 0.001	182/370	< 0.001
Score weighted by individual hormone associations^d,e^				
Quintile 1	42/134	1.0	20/74	1.0
Quintile 2	43/100	1.3 (0.8 to 2.2)	27/74	1.3 (0.7 to 2.6)
Quintile 3	40/71	1.8 (1.0 to 3.0)	35/74	1.7 (0.9 to 3.2)
Quintile 4	34/39	2.7 (1.5 to 4.8)	44/74	2.2 (1.1 to 4.0)
Quintile 5	23/26	2.9 (1.4 to 5.6)	56/74	2.7 (1.5 to 5.1)
Per 1 SD increase^d^	182/370	1.50 (1.24 to 1.81)	182/370	1.53 (1.25 to 1.87)
*P *trend	182/370	< 0.001	182/370	< 0.001

IGF-1 = insulin-like growth factor 1. ^a^Hormones included in the score are estrone, estradiol, estrone sulfate, testosterone, androstenedione, dehydroepiandrosterone, dehydroepiandrosterone sulfate and prolactin, then adding IGF-1 and c-peptide. ^b^RR and 95% CI were adjusted for age at blood draw and menopause, parity, history of benign breast disease, family history of breast cancer, fasting status and time of day of blood draw. ^**c**^Score is the number of hormones for which the participant had higher than the age- and assay batch-adjusted geometric mean hormone level. Among controls, the score with sex hormones and prolactin mean = 4.1 (SD = 2.4) and the score including IGF-1 and c-peptide mean = 5.0 (SD = 2.4). ^d^Score is the sum of the log_2_-transformed hormone level multiplied by the β coefficient for that hormone with breast cancer risk. Among controls, the score with sex hormones and prolactin mean = 10.8 (SD = 1.1) and the score including IGF-1 and c-peptide mean = 12.1 (SD = 1.2). ^e^Quintile cutpoints based on hormone score including all hormones.

## Discussion

We evaluated the combined influence of multiple sex and growth hormones on the risk of postmenopausal breast cancer on the basis of several scores. The scores ranked women by the number of hormones above the age- and batch-adjusted geometric mean and weighted the hormone values by their individual associations with breast cancer risk or proliferation of MCF-7 breast cancer cells. In each instance, there was a positive linear relationship between the score and risk of breast cancer overall and ER-positive disease. The risks in the highest categories were about double those in which only one hormone at a time was considered. When considering separate scores for estrogens and androgens, the estrogens had a slightly stronger association.

Very few studies have evaluated the relationship between multiple hormones simultaneously and breast cancer risk, and those that have considered interactions between two hormones generally have not revealed significant multiplicative interactions [2,5,7,8]. However, additive models were not considered. Trichopoulos *et al. *[9] measured estrone, estradiol, androstenedione, DHEAS, testosterone and IGF-1 in 29 prospective cases and 58 controls. The authors constructed a score summing the number of hormones with levels above the age-adjusted mean and observed that women with four to six versus one to three hormones above the mean did not have a different risk of breast cancer (RR = 1.13, 95% CI = 0.43 to 3.00, *P *trend = 0.53). Women with no hormones above the mean had a substantially lower risk of breast cancer compared to all other women (RR = 0.11, 95% CI = 0.01 to 0.90), suggesting that sex hormones may act as permissive factors for breast cancer growth. In contrast, our results do not support this hypothesis, as we observed a strong linear trend as the number of hormones above the geometric mean increased, which was more pronounced for ER-positive disease, although we also noted that women with no hormones above the mean versus any had a lower risk of breast cancer (RR = 0.55). Our results are similar regardless of how the hormone score was defined, in part because of the high correlation between the scores. The difference in results between the two studies likely stems from the greater statistical power in our study, since when we created a score using the same hormones as Trichopoulos *et al. *[9], we observed a linear association (RR, one-unit increase in number of hormones above the mean = 1.18, 95% CI = 1.07 to 1.31, *P *trend = 0.001).

Estrogens and prolactin likely influence the risk of breast cancer by inducing cell proliferation and tumor growth through the ER and prolactin receptor, respectively [32,33]. On the other hand, androgens have been hypothesized to increase breast cancer risk either directly by increasing cellular growth and proliferation or indirectly via conversion to estrogens [34]. In *in vitro *studies, androgens have been shown to either increase or decrease cell proliferation, depending upon the model system used [34]. Biological data have examined the combined effect of hormones. For example, in an Nb rat model, the simultaneous administration of testosterone and estradiol led to the development of invasive mammary carcinoma [35,36], but neither hormone alone induced carcinogenesis. Furthermore, in MCF-7 cells, both DHEA [24] and DHEAS [20] can increase proliferation independently of aromatase activity or binding of the ER. Similarly, in a breast cancer cell line induced to endogenously produce high levels of prolactin, proliferation was magnified by the addition of estradiol [37]. These biological studies suggest that hormones could act synergistically or through different cellular mechanisms (not only through the ER) to increase proliferation and ultimately breast carcinogenesis. Our results generally support this hypothesis in that the risk of breast cancer increased as the hormone scores increased.

We also observed that estrogens may have a slightly stronger influence on breast cancer risk than androgens. Our results are consistent with those of most prior studies [2-4], which have shown that when estradiol and testosterone (correlation approximately 0.30 to 0.35) are placed in the same model, the association with estradiol was essentially unchanged, but the association with testosterone was attenuated. However, investigators who conducted a combined analysis of nine prospective studies comprising 663 cases (including the cases in the present analysis) reported that estradiol and testosterone have independent associations from the other [1]. A complexity in the pooled analysis is that some studies used indirect assays to measure estradiol and testosterone, whereas others used direct assays, potentially leading to different correlations between these hormones across studies. The differences between studies may be due to sample size, different correlations between hormones examined or, in our study, consideration of multiple estrogen and androgens simultaneously. Additional research is needed to resolve the independent role of androgens and estrogens, as well as to determine the mechanism of action for androgens.

When considering the hormone score counting the number of hormones above the mean, the addition of IGF-1 and c-peptide increased the relative risk for the top category; however, this result was not replicated in the score weighted by individual hormone associations, possibly due to different reference groups. Researchers in two prior studies [7,8] observed that the risk of breast cancer was highest among women with high levels of both IGF-1 and at least one sex hormone (for example, testosterone or estradiol). Also, proliferation of MCF-7 cells was two times higher among cells treated with both IGF-1 and estradiol than when treated with IGF-1 or estradiol alone [38]. Although more studies are needed, it is possible that that IGF-1 and/or c-peptide, though not strongly associated with breast cancer risk individually, may be associated with a higher risk when considered in combination with other hormones.

This study has several strengths and limitations. First, case-control sets were assayed together, which reduces variability in hormone measures, and the assays had excellent coefficients of variation. Second, we had only one measure of each hormone to reflect long-term exposure; however, the intraclass correlations (ICCs) over two to three years were greater than 0.60 (except for prolactin, with an ICC of approximately 0.45) [39], suggesting good reproducibility over time. Importantly, in the same reproducibility study, the ICCs of the three hormone scores in our study ranged from 0.72 to 0.85 over two to three years, and the ICCs of the individual hormones generally were similar for women with high versus low hormone scores at the baseline blood draw. Third, our study was relatively large. Although this increased the study's power to detect statistically significant associations, our sample size for subanalyses (for example, interactions based on PMH use) was limited and as such we were not able to precisely assess whether a smaller subset of hormones could capture most of the variation across all hormones or directly compare associations for ER-positive versus ER-negative tumors. Not all the assays were measured for some women, although this was a random loss. When women with one missing hormone were included in the analysis (using the median level in the population for the missing hormone), the results were similar (data not shown). Fourth, although the score using the PI_m _was based on independent biological data, the score has some limitations. Since there was not a single study comparing all hormones, we drew data from multiple studies that used different metrics of proliferation and may have used different strains of MCF-7 cells. However, we standardized measures for each individual hormone by the comparable assay results for estradiol and averaged results across multiple studies. Also, other metrics, such as apoptosis or binding affinity to ER, would have been of interest but were not available for all hormones. Finally, some potential hormones of interest, such as melatonin and SHBG, either were not measured or had many missing values.

## Conclusions

Overall, this study suggests that having multiple sex and growth hormones with high circulating levels substantially increases the risk of breast cancer, particularly ER-positive disease. It has been hypothesized that the addition of estradiol and/or testosterone levels may improve risk prediction models for breast cancer [40]. Our results suggest that investigators in future studies should consider including these and other hormones when evaluating such models, although clearly a large sample size would be required because of the high correlations between hormones. Further research with increased sample sizes and longer follow-up time is necessary to confirm our findings, evaluate which subset of hormones may explain variation across all hormones, conduct testing of risk prediction model improvement, identify characteristics of women with low (or high) levels of many hormones, and better understand the biological interrelationships between hormones and breast cancer risk.

## Abbreviations

BMI: body mass index; DHEA: dehydroepiandrosterone; DHEAS: dehydroepiandrosterone sulfate; ELISA: enzyme-linked immunosorbent assay; ER: estrogen receptor; IGF-1: insulin-like growth factor 1; NHS: Nurses' Health Study; PMH: postmenopausal hormone use; RIA: RIA.

## Competing interests

The authors declare that they have no competing interests.

## Authors' contributions

All authors have made substantive contributions to this study, including its conception (SST and SEH) and design (SST, BAR, WCW and SEH) as well as the acquisition of data (SST and SHE) and the analysis and interpretation of the data (SST, BAR, WCW and SEH), and in the drafting of the manuscript (SST) or revising it critically for important intellectual content (BAR, WCW and SEH).
